# Anatomy-Guided Radiology Report Generation With Pathology-Aware Regional Prompts

**DOI:** 10.1109/OJEMB.2026.3687122

**Published:** 2026-04-23

**Authors:** Yijian Gao, Dominic Marshall, Xiaodan Xing, Junzhi Ning, Congren Dai, Giorgos Papanastasiou, Guang Yang, Matthieu Komorowski

**Affiliations:** Department of ComputingImperial College London 4615 SW7 2AZ London U.K.; Department of Surgery and CancerImperial College London 4615 SW7 2AZ London U.K.; Bioengineering Department and Imperial-XImperial College London 4615 W12 7SL London U.K.; Archimedes UnitAthena Research Centre 15125 Athens Greece; Bioengineering Department and Imperial-XImperial College London 4615 W12 7SL London U.K.; National Heart and Lung InstituteImperial College London 4615 SW7 2AZ London U.K.; Cardiovascular Research CentreRoyal Brompton Hospital 156726 SW3 6NP London U.K.; School of Biomedical Engineering & Imaging SciencesKing's College London 4616 WC2R 2LS London U.K.

**Keywords:** Anatomy detection, prompt-guided decoding, pathology localization, radiology report generation

## Abstract

*Goal:* Radiology report generation holds significant potential to alleviate clinical workloads and streamline medical care. However, achieving high clinical accuracy remains challenging, as radiographs often feature intricate structures and subtle pathologies. *Methods:* To address these challenges, this work introduces an innovative approach that explicitly integrates anatomical and pathological information into report decoding by leveraging pathology-aware regional prompts. Specifically, we develop an anatomical region detector that extracts structured visual features from distinct anatomical areas, coupled with a novel multi-label pathology detector that identifies global abnormalities. *Results:* Our model demonstrates superior report generation performance in natural language generation and clinical efficacy, surpassing previous state-of-the-art methods. It achieved scores of 0.394 in BLEU-1, 0.302 in ROUGE-L, and 0.470 in F1, reflecting substantial improvements in both linguistic fluency and medical accuracy. Formal expert evaluations further affirmed the model's potential to elevate radiology practice. *Conclusion:* By integrating anatomical and pathological insights to emulate radiologists' workflow, our model achieves superior accuracy and clinical coherence of radiology reporting. It offers remarkable promise to support clinical decision-making and transform patient management.

## I. Introduction

Every year, the demand for chest radiographs and their interpretation surpasses the growth of the radiology workforce. As the most common imaging modality, chest X-rays (CXR) account for nearly 2 billion exams annually worldwide [1]. This escalating overload often leads to reporting delays, forcing clinicians to make critical decisions without specialist input. Therefore, a well-optimized radiology reporting system holds immense potential to streamline workflows and enhance the efficiency of medical care.

Most radiology report generation studies have adopted the popular encoder-decoder workflow, which employs a visual encoder to extract image features, followed by a language decoder to generate free-text diagnostic reports [2]. Nevertheless, achieving high clinical accuracy and comprehensive coverage remains challenging [2], [3], primarily due to the intrinsic characteristics of radiological images (e.g., subtle lesions) and the complexity of lengthy reports.

Specifically, most existing works [4], [5], [6] rely on patch-level feature extraction with fixed grids in visual encoders, neglecting the inherent anatomical structures of radiographs and hampering the integration of holistic diagnostic views. This limitation frequently leads to incomplete and inconsistent reports, including redundant narratives of the same area, laterality errors, or insufficient differentiation between organs.

To address this issue, recent studies [7], [8], [9] have transitioned from patch-level to anatomical region-level encoders to facilitate the recognition of structured patterns with radiological semantics. For instance, Tanida et al. [7] proposed a region-guided report generation (RGRG) model, leveraging Faster R-CNN [10] to detect anatomical regions and modified GPT-2 Medium [11] to generate region-specific sentences, achieving strong completeness and interpretability. However, by grounding each sentence to a separate region, such approaches may introduce contradictory descriptions that risk clinical misinterpretation and weaken report coherence.

Beyond visual encoding, conventional language decoders, which primarily process only extracted visual features, often fail to explicitly harness the high-level pathological semantics of radiological images [7], [12]. As a result, clinically important abnormalities may be under-represented or inconsistently described in the generated reports.

Consequently, recent advances have utilized prompt learning to inject diagnostic semantics. PromptMRG [4] leveraged diagnostic-driven prompts derived from disease classification to guide report generation, with image features extracted by a ViT-based patch-level encoder. Their approach also incorporates report retrieval, achieving strong CE and competitive NLG metrics. Additionally, Wang et al. proposed a similar PromptRRG approach [13], which employed disease-enriched prompts generated from fixed templates and classification results. While prompt learning is effective in conveying domain knowledge [14], these methods mainly rely on image-level disease labels to construct the prompt and do not explicitly associate pathological findings with anatomical landmarks.

Recently, radiology report generation has been advanced by Vision-Language Models (VLMs) with pre-trained image encoders and fine-tuned large language model (LLM) decoders [3]. Methods such as CheXagent [15], MedDr [16] and XrayGPT [17] demonstrate promising multimodal understanding ability across various tasks. However, their effectiveness varies substantially across model architectures, data scales, and training strategies. In practice, limited high-quality image-report pairs and the risk of hallucinated clinical statements remain important challenges.

To bridge the gap between anatomical structure and pathological semantics in radiology report generation, we introduce a unified approach: *pathology-aware regional prompts*, which guide the decoder by injecting region-specific pathological information. Instead of relying on patch-level features, we develop an anatomical region detector as the encoder to extract structured visual features from distinct regions, effectively capturing both scale variations and clinically meaningful semantics in CXRs. In parallel, we introduce a novel multi-label pathology detector that identifies multiple abnormalities within a single area, accommodating the inherent spatial overlap of pathologies in CXRs. Detected pathologies are mapped to overlapping anatomical regions to construct pathology-aware regional prompts, which provide fine-grained diagnostic guidance for decoding. This design reflects radiologists' standard diagnostic practices, where anatomical regions are systematically assessed and pathological findings are associated with their locations before forming a comprehensive impression. Such integration enables coherent reasoning and diagnostically grounded report generation. We summarize our contributions as follows:

• We propose a novel radiology report generation system that reflects radiologists' diagnostic workflow by integrating anatomical structures and pathology findings via pathology-aware regional prompts, enabling clinically precise and coherent reports.
• We develop an anatomical region detector as the visual encoder to extract multi-scale, anatomy-grounded visual features from CXRs.
• We introduce a multi-label pathology detector that identifies multiple abnormalities within a single bounding box.
• Extensive experiments on MIMIC-CXR-JPG and Chest ImaGenome demonstrate that our model outperforms previous state-of-the-art methods in both language generation and clinical efficacy, with diagnostic quality further supported by expert evaluation.

## II. Materials and Methods

### A. Pipeline Overview

**Figure 1.**
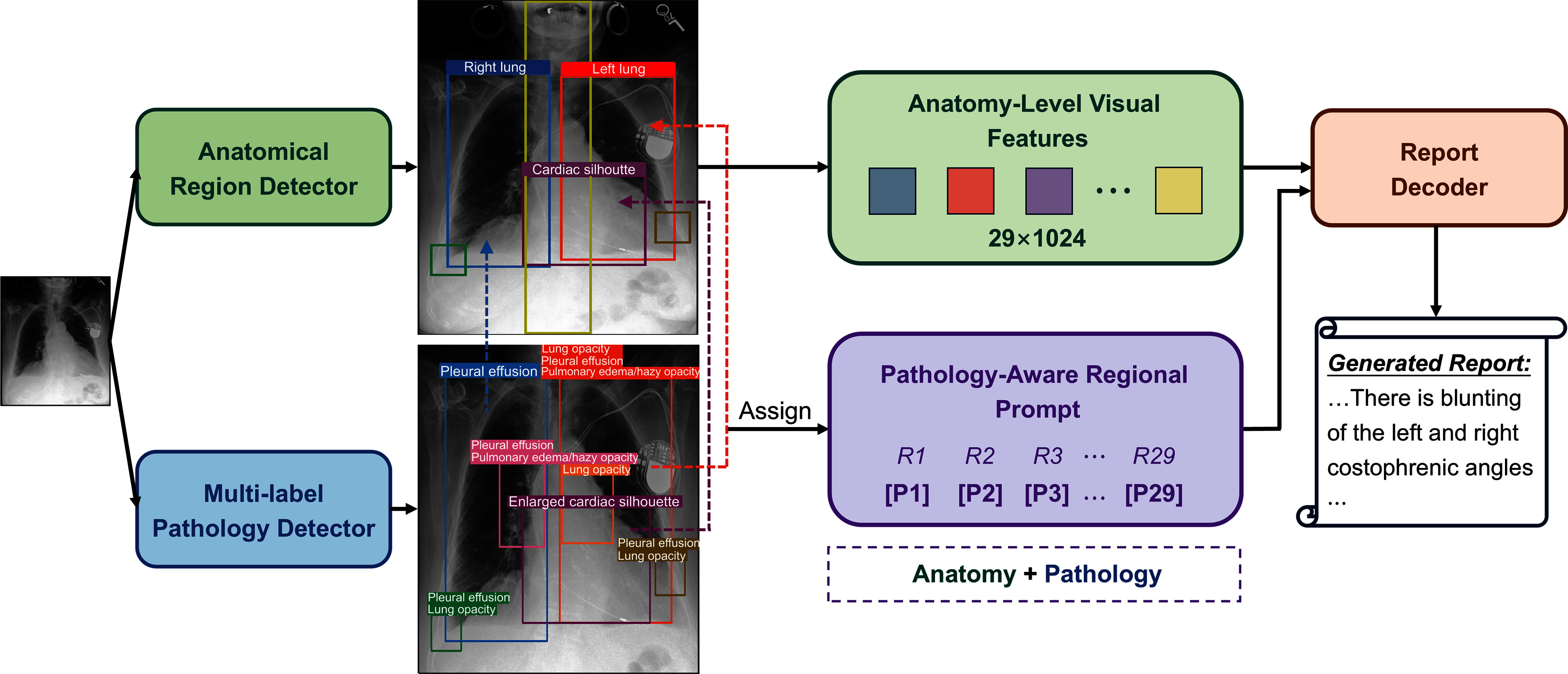
Model pipeline. The anatomical region detector first identifies and extracts visual features from 29 region bboxes, with the first six illustrated. Meanwhile, the multi-label pathology detector locates global abnormalities, capturing multiple findings per bbox. A 29-token prompt $T$ is then formed by assigning each region $R_{i}$ a corresponding pathology token $P_{i}$ based on spatial bbox overlap. Finally, the report decoder is guided by both the anatomy-level visual features and the pathology-aware regional prompt to generate reports.

As illustrated in Fig. 1, our pipeline begins with an anatomical region detector that identifies 29 predefined regions and extracts corresponding visual features, providing a structured anatomy-level visual context to the decoder. Meanwhile, a novel multi-label pathology detector locates multiple abnormalities at the image level, capturing high-level pathological semantics. The outputs from both detectors are then associated based on their spatial overlaps to construct pathology-aware regional prompts, which explicitly provide region-specific pathological insights. Finally, the report decoder employs both the anatomy-level visual features and the prompt guidance to produce medically relevant descriptions of the CXR. The pipeline is trained in two stages and can perform inference end-to-end. We first train the anatomical and pathology detectors separately, and then freeze them to train the full report generation model.

### B. Anatomical Region Detector

This module serves as the visual encoder, aimed at locating 29 distinct anatomical regions in a CXR. We utilize Faster R-CNN [10] with a ResNet-50 [18] backbone pre-trained on ImageNet [19] to extract preliminary features. These features are processed by a Region Proposal Network (RPN) to generate object proposals, represented as bounding boxes (bbox):

\begin{equation*}
r_{k} = (r_{k,x1}, r_{k,y1}, r_{k,x2}, r_{k,y2}), k=1,2,\ldots,29, \tag{1}
\end{equation*}

where $k$ indexes each proposal, and $(x_{i}, y_{i})$ are the top-left and bottom-right bbox coordinates.

Following [7], we identify top' region proposals and employ a Region of Interest (RoI) pooling layer to produce uniformly sized feature maps, noted as $R_{k}\in \mathbb {R}^{2048 \times H \times W}$. A subsequent 2D average pooling followed by a linear transformation reduces the spatial dimensions, yielding anatomy-level feature vectors $R \in \mathbb {R}^{29 \times 1024}$, which are used for decoding.

### C. Multi-Label Pathology Detector

This module enhances the encoding stage by detecting global pathologies in CXRs. Since radiographs frequently exhibit multiple pathologies at the same anatomical site, we perform multi-label training by introducing a label squeeze function into the YOLOv5x model [20]. Specifically, bboxes that share identical coordinates but have different class labels are merged into a single entry with a multi-hot class vector, and the bbox classification loss is computed using Binary Cross-Entropy over all pathology classes. YOLOv5x is used for its efficient one-stage design, straightforward adaptation to the multi-label setting, and its advantage on small object detection [21].

Furthermore, to alleviate the long-tail label distribution in the Chest ImaGenome dataset [22] and improve training stability, we perform class reduction by removing rare pathology classes constituting less than 0.5 of the dataset. This cutoff is consistent with clinical observations that extremely rare findings often have limited report-level impact. As a result, the original 42 pathology classes are reduced to a concise set of 21 representative findings. Subsequently, we refine bbox labels by removing overly broad parent classes when more specific descendants are present in the pathology hierarchy specified in the official Chest ImaGenome ontology, as illustrated in Supplementary Fig. 2 (e.g., removing the root node lung opacity when a more specific child finding exists). This refinement reduces the imbalance introduced by high-frequency coarse labels. Together, these steps yield a more balanced and discriminative pathology set for multi-label detection. (See Supplementary Materials for details of the strategy of label squeeze, class reduction and hierarchy-based label refinement.)

**Figure 2.**
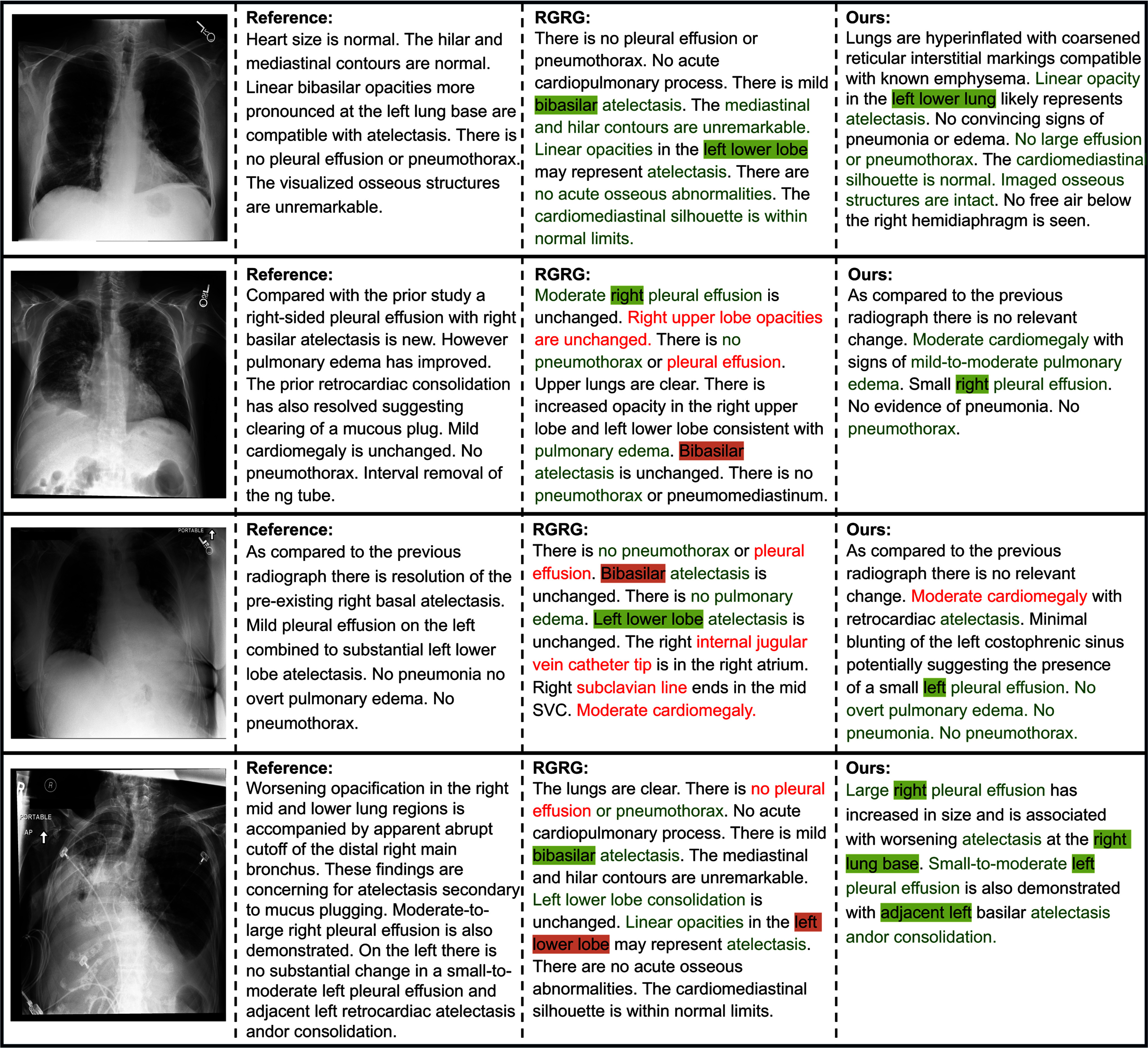
Examples of reports generated by RGRG [7] and our model. Green font indicates correct descriptions, while red font denotes incorrect mentions of negative or unmentioned findings. Green highlight confirms correct pathology locations and red highlight indicates errors.

### D. Pathology-Aware Regional Prompts

To associate the extracted anatomical regions and pathological information to guide the report decoder, we introduce pathology-aware regional prompts. A prompt $T \in \mathbb {R}^{29 \times 1}$ is formulated as a sequence of 29 pathology tokens $\lbrace P_{1}, P_{2}, \ldots, P_{29}\rbrace$, where each token $P_{i}$ corresponds to the pathological finding associated with the anatomical region $R_{i}$.

During training, ground truth prompts are directly constructed from scene graphs provided in the Chest ImaGenome dataset [22], which explicitly annotate the associations between anatomical regions and pathological findings. Each anatomical region is assigned a single pathology token using a rule-based strategy to avoid ambiguity from overlapping or hierarchical labels. During inference, prompts are dynamically generated by associating detected pathologies with anatomical regions based on the highest Intersection over Union (IoU) between their respective bounding boxes, as illustrated by the dashed lines in Fig. 1. For each region, the most representative detected pathology is selected using the same assignment principles as in training. (See Supplementary Materials for detailed assignment rules.)

### E. Report Decoder

This module generates diagnostic reports by jointly decoding anatomy-level visual features and textual guidance. During training, the ground truth prompt is prepended to the reference report to provide supervision, enabling the decoder to learn cross-modal associations between textual prompts and visual features. During inference, the dynamically generated prompts are provided as textual guidance to support report generation.

### F. Evaluation Metrics

*a) Automated metrics:* We evaluate the model performance using Natural Language Generation (NLG) and clinical efficacy (CE) metrics. NLG metrics, including BLEU-n [23], METEOR [24], and ROUGE-L [25], assess textual similarity between generated reports and reference reports. CE metrics, including Precision, Recall, and F1-score based on findings extracted by the CheXbert labeler [26], evaluate the accuracy of medical observations.

*b) Expert evaluation metrics:* Assessing radiology reports is challenging due to complex interpretation and subtle clinical language. To fully capture the appropriateness of key details or the risks posed by redundant information, we establish clinically relevant metrics with certified medical experts (M. Komorowski and D. Marshall) to support formal expert evaluation (See Supplementary Materials for details):

1) *Rubric (15):* Comparative rating of two reports based on overall quality.
2) *Brevity:* Assessment of whether the report is overly concise (-1) or verbose (+1).
3) *Accuracy (15):* Independent rating of diagnostic correctness and clinical detail.
4) *Danger (0/1):* Binary judgment of whether the report could lead to serious clinical harm.

## III. Results

### A. Datasets

We utilize the Chest ImaGenome v1.0.0 dataset [22], built upon MIMIC-CXR-JPG [31], [32] that contains image-report pairs from around 65,000 patients. Chest ImaGenome augments MIMIC with scene graphs for each frontal X-ray, annotating bounding boxes for 29 anatomical regions and associated pathologies. Follow the official dataset split and discarding samples without a *Findings* section, we obtain 113,915 training, 15,658 validation, and 32,711 test samples.

### B. Automated Metrics Comparison

The performance of our model is evaluated against various SOTA baselines, including R2Gen [28], R2GenCMN [6], Clinical-BERT [29], METrans [12], CAMANet [5], RGRG [7], PromptMRG [4], and VLM-based approaches such as XrayGPT [17], MedDr [16], CheXagent [15] and AdAMatch-Cyclic [30], which employ pre-trained LLM decoders. Table 1 demonstrates that our model achieves the best overall results, outperforming SOTA baselines in 4 out of 6 NLG metrics and 2 out of 3 CE metrics, with the largest gains in BLEU-1, ROUGE-L, Precision and F1-score.

**TABLE 1:** Comparison With State-of-the-Art Methods on the MIMIC-CXR Dataset Using NLG and CE Metrics. * Denotes Methods Re-Implemented and Tested by Us Following Official Implementations. - Indicates Metrics Not Reported in the Original Publications. The Performance of XrayGPT Is Referenced from [27]. All Other Results Are Sourced From Original Publications. BL, MTR, and RG-L Represent BLEU, METEOR, and ROUGE-L, Respectively.

Model	NLG metrics							CE metrics		
		BL-1	BL-2	BL-3	BL-4	MTR	RG-L		Precision	Recall	F1
R2Gen [28]	0.353	0.218	0.145	0.103	0.142	0.277		0.333	0.273	0.276
R2GenCMN [6]	0.353	0.218	0.148	0.106	0.142	0.278		0.334	0.275	0.278
ClinicalBERT [29]	0.383	0.230	0.151	0.106	0.144	0.275		0.397	0.435	0.415
METrans [12]	0.386	0.250	0.169	0.124	0.152	0.291		0.364	0.309	0.311
XrayGPT [17]	0.128	0.045	0.014	0.004	0.079	0.111		-	-	0.326
CAMANet [5]	0.374	0.230	0.155	0.112	0.145	0.279		0.483	0.323	0.387
RGRG* [7]	0.373	0.249	**0.175**	**0.126**	0.168	0.264		0.461	**0.475**	0.447
AdaMatch-Cyclic [30]	0.379	0.235	0.154	0.106	0.163	0.286		-	-	-
MedDr [16]	0.322	-	-	0.072	**0.238**	0.226		-	-	-
CheXagent* [15]	0.200	0.123	0.083	0.058	0.104	0.249		0.477	0.273	0.348
PromptMRG* [4]	0.387	0.230	0.147	0.100	0.148	0.260		0.505	0.461	0.453
Ours	**0.394**	**0.251**	0.173	**0.126**	0.151	**0.302**		**0.509**	0.437	**0.470**

RGRG achieves the second-best performance overall by generating region-specific descriptions for selected regions. Our model improves upon this by utilizing anatomy-level visual features for all regions and integrating pathology-aware regional prompts, improving most metrics with a notable 2.3 increase in F1-score. Furthermore, PromptMRG performs strongly with image-level prompting but covers only 15 disease classes without disease localization. Our model detects 21 pathologies and correlates them with specific anatomical regions, achieving consistent gains in all metrics with a significant 1.7 increase in F1-score and the highest Precision. Overall, these comparisons support the effectiveness of anatomypathology alignment for improving clinically relevant reporting while limiting false-positive mentions.

Table 1 also reveals considerable variance among VLMs with pre-trained LLM decoders. XrayGPT reports notably lower NLG scores, possibly due to limited domain-specific image-report alignment. CheXagent shows relatively modest NLG scores, whereas AdaMatch-Cyclic attains competitive performance, suggesting that its fine-grained image-text alignment cues like patch-word associations can be beneficial. Overall, these observations indicate that alignment design and domain adaptation can play a larger role in VLM-based report generation than using fintuned LLM decoder alone.

### C. Formal Expert Evaluation

In the expert evaluation, our model is compared against three baselines: RGRG [7], PromptMRG [4], and CheXagent [15]. The first two models employ enhanced encoder-decoder architectures, while CheXagent is a large-scale Vision-Language Model (VLM).

The results are summarized in Table 2. Our model achieves the best conciseness with a Brevity score of 0.01 and the lowest Danger score of 0.03, indicating fewer potentially harmful errors under expert review. It also performs comparably well in Rubric and Accuracy, showing close agreement with reference reports. CheXagent attains the highest Accuracy score of 3.55 but can be overly verbose, as reflected by its Brevity score. PromptMRG shows strong adherence to clinical guidelines, with a slightly higher Rubric score than our model, but performs less favourably on Brevity and Danger. RGRG ranks second in automated metrics but shows weaker expert-evaluated performance, including the highest Danger score.

**TABLE 2:** Comparison of Formal Expert Evaluation Results. **$\uparrow$**: Higher Is Better; **$\downarrow$**: Lower Is Better; **$\bigtriangleup$**: Absolute Value.

Model	Rubric $\uparrow$	Brevity $\bigtriangleup \downarrow$	Accuracy $\uparrow$	Danger $\downarrow$
CheXagent [15]	2.28	0.08	**3.55**	0.03
PromptMRG [4]	**2.32**	0.19	3.49	0.03
RGRG [7]	2.21	0.40	3.23	0.14
Ours	2.26	**0.01**	3.51	**0.03**

To investigate the origin of the dangerous errors, a brief review of the flagged cases was conducted. The results suggest that such errors mainly stem from incorrect descriptions or mislocalization of common but clinically sensitive findings. Additionally, suboptimal image quality, such as poor patient positioning, can exacerbate such failures, while dangerous errors may still occur under standard imaging conditions.

### D. Instance Performance

Fig. 2 presents several examples comparing the reports generated by our model and RGRG [7], which is the second-best baseline in automated metrics. Overall, our model more consistently captures key pathological findings and produces reports that are closely aligned with the reference report.

Moreover, our model accurately identifies the presence, extent, and location of pathologies. In the last row of Fig. 2, it precisely reports a large right-sided and a small-to-moderate left-sided pleural effusion. In contrast, RGRG overlooks them and incorrectly reports linear opacities in the opposite lung, likely due to the mirror effect in radiographs that reverses laterality. It also hallucinates device-related pathologies, as shown in the third row. Notably, RGRG can produce contradictory statements. For instance, in the second row, it correctly describes a moderate right pleural effusion but later denies any effusion in the same image. Such inconsistencies may mislead clinical decisions and contribute to diagnostic errors or delays.

These failures are consistent with the design of RGRG, where a region selector may omit salient findings and region-wise sentences are generated independently without cross-region context. The lack of explicit pathology cues can also increase omissions and spurious findings. Our model addresses these by integrating anatomy-level features with detected pathological cues, providing consistent region-wise guidance during decoding for improved report coherence.

### E. Ablation Study

To evaluate the effectiveness of our pathology-aware regional prompts, an ablation study is conducted. In the baseline setting, the report decoder is initialized with a predefined start token and is conditioned only on anatomy-level visual features during training and inference. We then add prompt guidance to the baseline as textual conditioning to the decoder input, which are constructed according to the region-pathology associations defined in Section II-D.

**Figure 3.**
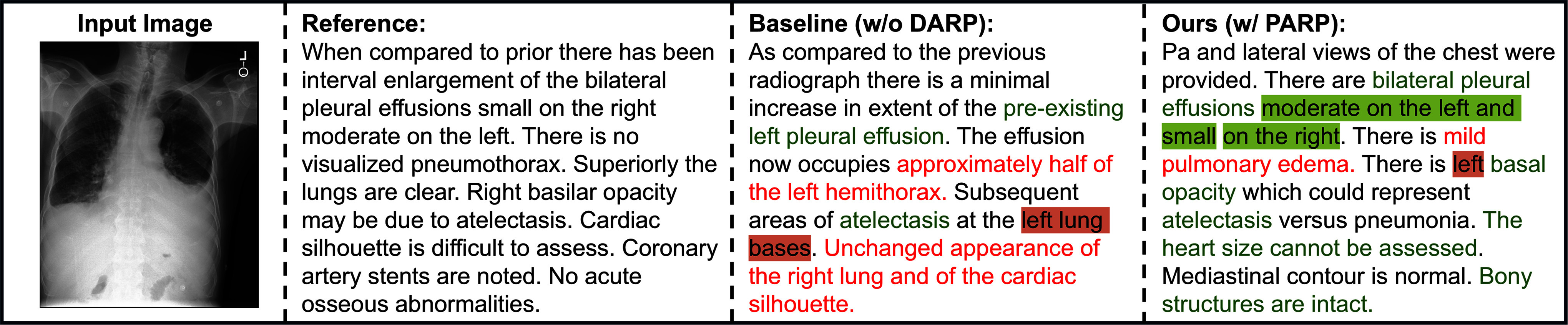
Ablation study examples. Green font indicates correct descriptions, while red font denotes incorrect mentions of negative or unmentioned findings. Green highlight confirms correct pathology locations and red highlight indicates errors.

As shown in Table 3, incorporating pathology-aware regional prompts leads to improvements across all NLG and CE scores. Specifically, the F1-score increases by 8.9, highlighting the crucial role of prompt guidance in producing diagnostically accurate reports. Moreover, Fig. 3 presents qualitative comparisons between the baseline and our model, which are consistent with the quantitative results. Specifically, the baseline identifies key findings like left pleural effusion but misreports its extent. With pathology-aware regional prompts, our model more accurately detects and describes critical diagnostic details, correctly noting pleural effusions as moderate on the left and small on the right without false positives. This qualitative alignment further validates the effectiveness of our proposed approach.

**TABLE 3:** Ablation Study Comparing Baseline and Our Model on NLG and CE Metrics. PARP Denotes Pathology-Aware Regional Prompts.

Model	NLG metrics							CE metrics		
		BL-1	BL-2	BL-3	BL-4	MTR	RG-L		P	R	F1
Baseline (w/o PARP)	0.364	0.237	0.166	0.122	0.141	0.300		0.464	0.323	0.381
Ours (w/ PARP)	**0.394**	**0.251**	**0.173**	**0.126**	**0.151**	**0.302**		**0.509**	**0.437**	**0.470**

## IV. Discussion

Experimental evidence consistently validates the effectiveness of integrating anatomy-grounded features with pathology-aware regional prompts for radiology report generation. Across automated benchmarks, our method achieves the best overall performance, suggesting gains beyond textual similarity toward finding-level clinical correctness. The ablation study further confirms that prompt guidance is a key contributor to these gains. Instance-level comparisons demonstrate that our model more reliably preserves laterality and produces fewer clinically implausible or contradictory statements, which are clinically salient failures. Formal expert evaluation further indicates that our model achieved improved conciseness and safety while maintaining clinical quality.

However, absolute automated metrics on this task remain modest, reflecting the intrinsic challenge of radiology report generation and its evaluation. Standard n-gram-based NLG metrics mainly quantify text similarity to the reference report, which usually varies across observers and styles. Consequently, these metrics may underestimate clinical adequacy when key findings are correctly described but phrasing differs [2]. We thus complement NLG results with clinically grounded evaluations, including CE metrics and expert assessment, which more directly reflect diagnostic correctness.

Taken together with the instance-level analyses, our clinically grounded evaluations reveal that misidentifying fine-grained or visually similar abnormalities remains a source of clinically meaningful errors, suggesting that current systems remain far from fully reliable deployment. Furthermore, our current framework relies on Chest ImaGenome scene graphs which provide only frontal-view supervision and limit direct evaluation on multi-view datasets such as IU X-ray [33]. We also apply class reduction to stabilize multi-label training under long-tailed labels, which may reduce coverage of extremely rare findings. Therefore, our system is best positioned as a clinical assistant that produces initial drafts and structured clinical insights for revision, rather than autonomous operation.

Looking forward, bridging the remaining performance gap requires advances in data, learning, and evaluation. Larger and more diverse datasets with richer regionfinding supervision are essential for improving diagnostic fidelity and coverage, while long-tailaware learning strategies can help reduce clinically meaningful errors on rare but important findings. Broader clinical evaluation across institutions and patient populations is crucial for assessing real-world usability, together with improved interpretability and uncertainty awareness to support safe human-AI collaboration. More clinically aligned and practically implementable evaluation metrics are also needed to better reflect clinical correctness and utility. Together, these directions pave the way for clinically valuable and trustworthy report generation.

## V. Conclusion

This work presents a novel radiology report generation framework integrating anatomical and pathological information through pathology-aware regional prompts. By aligning region-level anatomical features with high-level pathology cues, our model provides structured and clinically grounded guidance for report decoding. Extensive automated and clinical-grounded evaluations demonstrate consistent improvements in clinically salient detail preservation, report coherence, and safety. Ablation results confirm the central role of pathology-aware prompt guidance in these gains. Future work will focus on extending the framework to more diverse data settings, improving robustness to distributional imbalance and rare clinical findings, and pursuing broader and more rigorous clinical validation to further assess real-world applicability.

## Supplementary Materials

Supplementary Materials present detailed methodologies, evaluation metrics, implementation details, additional experimental results, and an extended discussion on limitations and future work.

Supplementary Materials
