# Supplementary material for: Anatomy-Guided Radiology Report Generation With Pathology-Aware Regional Prompts
Source: IEEE Open J Eng Med Biol. 2026 Apr 23;7:165–71. doi: 10.1109/OJEMB.2026.3687122 (PMC13175599; doi:10.1109/OJEMB.2026.3687122)
Supplement: Supplementary Materials [file supp1-3687122.pdf]

Supplementary Materials

Anatomy-Guided Radiology Report Generation with Pathology-Aware Regional Prompts

Yijian Gao, Dominic Marshall, Xiaodan Xing, Junzhi Ning, Congren Dai, Giorgos Papanastasiou, Guang Yang*, *Senior Member, IEEE*, Matthieu Komorowski*

I. Materials and Methods

A. Multi-label Pathology Detector

1) Label Squeeze: Unlike traditional object detection tasks with single labels per bbox, CXRs often display multiple findings at the same location. To address this complexity, we design a label squeeze strategy to facilitate multi-label training.

Originally, the label vector l for a CXR image is shaped as $[N,5]$, representing N pathology bboxes. Each bbox is labeled as $\{class, bbox_i\}$, where $bbox_i = \{x,y,w,h\}$ denotes the bbox location. In the multi-label setting, a single bbox may contain several classes. Therefore, for each $bbox_i$, a class vector c_i of length C is defined, where C is the total number of possible classes. This vector is populated according to the set of class labels associated with $bbox_i$. Furthermore, bboxes that share the same coordinates but differ in class labels are merged into a single entry with a unique multi-hot label vector.

Consequently, the label vector per image l' is reshaped to $[M, C+4]$, where each entry $l'_i = \{c_i, bbox_i\}$, and M denotes the number of unique bboxes, each corresponding to a distinct spatial location within the image.

Therefore, the bbox classification loss for $bbox_i$ is given by:

$$L_{\text{cls}} = \sum_{i=1}^{M}\sum_{j=1}^{C} -\left(c_i[j]\cdot\log(\hat{c}_i[j]) + (1-c_i[j])\cdot\log(1-\hat{c}_i[j])\right), \quad (1)$$

where $\hat{c}$ is the predicted class vector.

The objectness loss L_{obj} measures the model's confidence in detecting any object within a bbox using Binary Cross-Entropy (BCE) loss between the predicted object presence score and the actual presence (1 for object presence and 0 otherwise). The bbox regression loss L_{box} calculates the accuracy of the predicted bbox coordinates compared to the ground truth using Mean Squared Error (MSE) loss for $bbox_i$. Finally, the total loss for the pathology detector combines these elements:

$$L = \lambda_{\text{cls}}L_{\text{cls}} + \lambda_{\text{obj}}L_{\text{obj}} + \lambda_{\text{box}}L_{\text{box}}, \quad (2)$$

with $\lambda_{\text{cls}}, \lambda_{\text{obj}}, \lambda_{\text{box}}$ as the respective weights for each loss component.

2) Class Reduction: The proposed pathology detector is developed on the Chest ImaGenome Dataset [1], which provides bbox coordinates and labels for 42 pathologies across anatomical regions in CXRs. However, as shown on the left of Fig. 1, the dataset exhibits an extremely imbalanced distribution, which significantly impairs computer vision tasks [2]. To alleviate the long-tail effect and prioritize crucial abnormalities before training, tail classes that constitute less than 0.5% of the training data were removed.

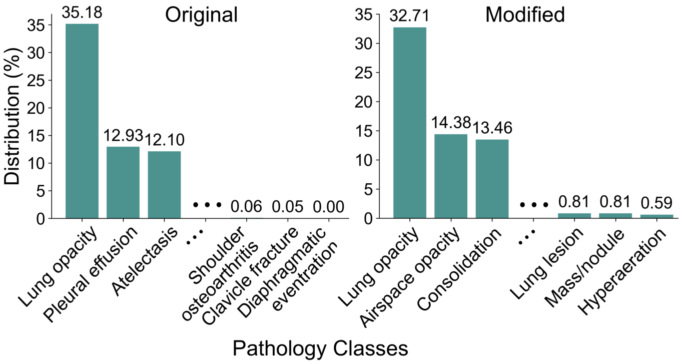

Fig. 1. Distribution of pathology classes, showing only the top three and bottom three classes by frequency. Original (42 classes); Modified (21 classes).

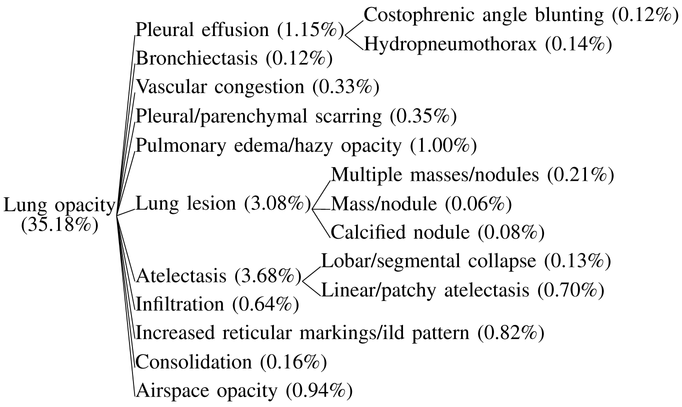

Fig. 2. Parent-child relationship adopted from the official Chest ImaGenome label ontology [1]. The hierarchy is visualized for clarity, with 'lung opacity' as the root node and its second- and third-level descendants shown along with their relative proportions in the dataset (in brackets). Sibling categories of 'lung opacity' without descendants are omitted.

3) Hierarchy-based Label Refinement: As illustrated in Fig. 2, the Chest ImaGenome dataset defines a hierarchical parent-child relationship for pathology classes, which can introduce label redundancies. For example, when a child node is annotated within an anatomical region, its parent nodes are also included, potentially biasing object detection models toward broader, less specific categories. Notably, the root node 'lung opacity' has the largest set of descendants and is therefore disproportionately represented.

To mitigate this bias, we remove the root node 'lung opacity' from the class vector of a bbox and retain only its more specific descendants whenever a third-level node is present. This modification, shown in Fig. 1, reduces the classes to 21,

thereby minimizing redundancy and alleviating the imbalance in pathology class distribution.

B. Pathology-Aware Regional Prompts

The construction of the proposed prompts during training and inference is described as follows.

1) Training: During training, the ground truth prompt for each image is derived from scene graphs that provide bbox coordinates of anatomical regions and their corresponding pathologies [1]. Specifically, we adopt a rule-based strategy to assign a pathology token label to each anatomical region. The rules are as follows:

1) If a region's pathology label has no child nodes or only contains 'lung opacity', it is assigned its own token.
2) If a second-level node appears with 'lung opacity', the second-level token is used due to its higher diagnostic specificity.
3) If a third-level node is present, we still assign the second-level token to balance specificity and detectability, as third-level nodes are rare and may cause inconsistencies between training and inference.
4) If multiple pathologies remain, the least frequent finding is retained to further mitigate the long-tail effect.

This systematic labeling strategy ensures that each anatomical region is associated with the most indicative and distinct disease token, facilitating precise and informative training of the report decoder.

2) Inference: During inference, detected pathologies are assigned to overlapping regions based on the Intersection over Union (IoU) between their bboxes. Specifically, a pathology bbox is assigned to a region if the IoU between them exceeds a certain threshold. If multiple bboxes overlap with the same region, the one with the highest IoU is selected. For bboxes with multiple findings, we employ the same rule-based token prompt construction as in training to assign each region a token.

If an anatomical region does not match any pathology bbox (i.e., falls below the IoU threshold or is not covered by any detection), the corresponding token is set to '[NEG]'.

C. Evaluation Metrics

1) Formal Expert Evaluation: The detailed definition and scoring guidelines of each metric are as follows:

1) **Rubric (1-5)**: Following Yang et al. [3], clinicians compare two radiology reports and offer enhanced granularity regarding patient impact by grading from X, B2, B1, C, A2 and A1 (maps to 1-5 in our experiments).
2) **Brevity**: Clinicians assess report verbosity by: Too Concise (-1), Good (0), and Too Verbose (+1).
3) **Accuracy (1-5)**: As defined in Table I, clinicians evaluate report quality in the absence of a good reference, focusing on disease identification, detail of findings, and impact on patient management [4].
4) **Danger (0/1)**: This metric collects clinicians' responses on whether a report is acutely dangerous, meaning it could pose an immediate and severe health risk. While an accuracy score of 1 typically accounts for such issues, explicitly flagging these critical errors is essential, as they represent major failures that could hinder the adoption of the model.

TABLE I. Accuracy Definition in Formal Expert Evaluation [4]

Score	Definition
5	Perfect report, accurately detailed
4	Generally accurate, a few missing details
3	Key details present but require additional interpretation with no issues regarding patient management
2	Missing key details but not dangerous
1	Dangerous (would lead to mismanagement)

The experts assessed generated reports from 100 randomly selected test samples. Moreover, to ensure fairness and minimize bias, model outputs were anonymized and presented in randomized order.

2) Object Detection: The performance of the anatomical region detector is assessed using the Intersection over Union (IoU) metric, defined as $\text{IoU} = \frac{\text{Area of Intersection}}{\text{Area of Union}}$. We report the mean IoU and average number of detected regions per image.

For assessing the multi-label pathology detector, we adopt standard object detection metrics, including Precision, Recall, and mean Average Precision (mAP) at IoU thresholds of 0.5 and 0.95, respectively.

D. Implementation Details

Our pipeline is trained in two stages using PyTorch 2.0 on a single NVIDIA RTX 4090 GPU.

1) Anatomical Region Detector: Images are resized to 512 pixels on the shorter side while maintaining aspect ratio, then cropped to 512×512. We employ random cropping during training and centre cropping for inference. The learning rate is initially set to 1e-3 and optimized using an AdamW [5] with a weight decay of 1e-2. Training runs for 10 epochs with a batch size of 16.

2) Multi-Label Pathology Detector: Images are resized to 640×640, and the model is trained for 80 epochs at a batch size of 50. During inference, the confidence threshold is set to 0.35 and the IoU threshold to 0.45 for non-maximum suppression. L_{cls}, L_{obj} and L_{box} are set to 0.5, 1.0, and 0.05, respectively.

Moreover, before training the multi-label pathology detector, we further refine the Chest ImaGenome dataset following the YOLOv5 official guide [6] to retain 12% of the empty background CXRs.

3) Full Model: We employ the BERT-base model [7] in decoder-only mode trained from scratch. To support prompt-based decoding, we add all possible pathology tokens to the tokenizer's vocabulary. The tokens for each pathological finding are shown in Table III.

The IoU threshold for assigning detected pathologies to anatomical regions is set to 0.4. The BERT model uses a hidden size of 1024, matching the dimension of the anatomy-level visual features R. An AdamW optimizer with a weight decay of 0.05 is used, and the initial learning rate of 5e-5 follows a cosine schedule. Beam search with a width of 4 is applied for sentence generation. Training is conducted for 15 epochs with a batch size of 14.

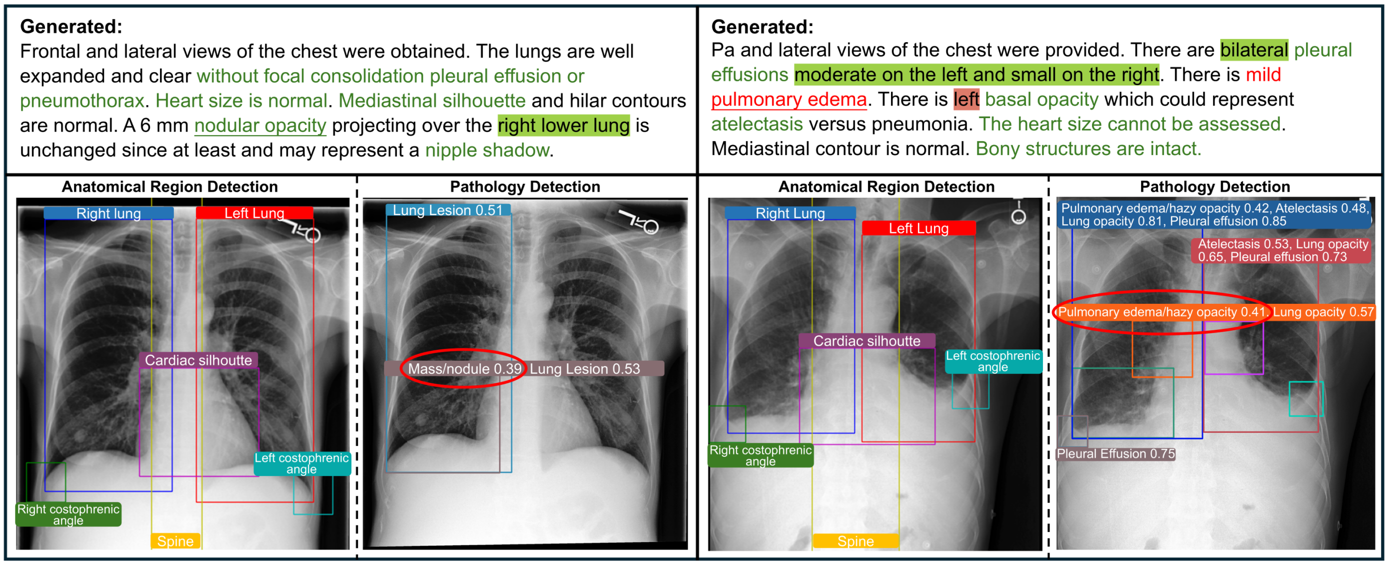

Fig. 3. Examples of generated reports and detection results. The anatomical region detection results include bboxes for 29 regions, with only the first 6 shown for clarity. Pathology bboxes are labelled with detected findings and their confidence scores. The red circle highlights detected findings that closely align with the corresponding description in the generated report.

II. Results

A. Pathology-Report Alignment

Existing report generation systems often lack interpretability and transparency, hindering their adoption in AI-aided diagnostics. Our model addresses this gap by leveraging the multi-label pathology detector that identifies findings at a global scale. These findings then serve as interpretable, intermediate outputs for tracing the generated content.

As depicted in Fig. 3, the generated report from our model exhibits strong alignment with the pathology detection results. For instance, in the left example, the detector accurately identifies a mass/nodule and its parent node, lung lesion, which are then assigned to the right lung for prompt construction. Consequently, the generated report correctly describes a nodular opacity in the right lung. Moreover, in the right example, a mild pulmonary edema is incorrectly reported by our model. This error can be traced back to the model mistakenly detecting this condition in the right lung, which subsequently propagated the erroneous information.

Such consistency between the generated report and detection results is crucial, particularly in cases of erroneous outputs or ambiguous findings. It offers transparency by enabling clinicians to pinpoint inaccuracies in the generated report, thereby significantly enhancing the clinical utility and trustworthiness of the automated system.

B. Detectors Performance

1) Anatomical Region Detector: Table II presents the micro-average IoU scores for all anatomical regions. Our model achieves an average IoU of 0.892 across all 29 regions, demonstrating its comprehensive accuracy. In addition, it consistently detects an average of 28.943 regions per image, indicating high precision in anatomical identification. We attribute this effectiveness in part to the well-distributed anatomical regions per image, which optimize model training.

TABLE II. Micro average IoU and detected regions per image of the anatomical region detector across all 29 regions.

Anatomical Region	IoU	Avg. Detection Regions
Right Lung	0.942	29.0
Right Upper Lung Zone	0.942	28.971
Right Mid Lung Zone	0.915	28.971
Right Lower Lung Zone	0.909	28.971
Right Hilar Structures	0.909	28.971
Right Apical Zone	0.903	28.942
Right Costophrenic Angle	0.916	28.942
Right Hemidiaphragm	0.862	28.855
Left Lung	0.944	29.0
Left Upper Lung Zone	0.944	28.971
Left Mid Lung Zone	0.922	28.971
Left Lower Lung Zone	0.897	28.942
Left Hilar Structures	0.907	28.971
Left Apical Zone	0.911	28.971
Left Costophrenic Angle	0.919	28.971
Left Hemidiaphragm	0.823	28.942
Trachea	0.879	29.0
Spine	0.955	29.0
Right Clavicle	0.884	28.826
Left Clavicle	0.879	28.913
Aortic Arch	0.846	28.913
Mediastinum	0.893	28.971
Upper Mediastinum	0.910	28.913
SVC	0.841	28.913
Cardiac Silhouette	0.867	28.971
Cavoatrial Junction	0.772	28.739
Right Atrium	0.837	28.913
Carina	0.783	28.913
Abdomen	0.947	29.0
Average	**0.892**	**28.943**

TABLE III. Pathology detector performance on standard object detection metrics. The corresponding token of each pathology is also demonstrated. Regions with no matching bbox (i.e., below the IoU threshold or not covered) are assigned the token '[NEG]'. P and R denote Precision and Recall.

Pathology	Token	Precision	Recall	mAP@0.5	mAP@0.95
Lung opacity	[LUN]	0.575	0.646	0.577	0.451
Airspace opacity	[AIR]	0.406	0.061	0.222	0.191
Consolidation	[CON]	0.352	0.075	0.203	0.176
Atelectasis	[ATE]	0.561	0.516	0.5	0.412
Linear/patchy atelectasis	[LPA]	0.351	0.121	0.207	0.195
Lobar/segmental collapse	[LSC]	0.376	0.054	0.21	0.154
Pulmonary edema/hazy opacity	[PEH]	0.493	0.549	0.478	0.369
Vascular congestion	[VAS]	0.406	0.328	0.294	0.244
Vascular redistribution	[VRE]	0.5	0.01	0.253	0.24
Pleural effusion	[PLE]	0.589	0.531	0.558	0.401
Costophrenic angle blunting	[CAB]	0.394	0.047	0.214	0.184
Pleural/parenchymal scarring	[PPS]	0.431	0.187	0.277	0.242
Enlarged cardiac silhouette	[ECS]	0.608	0.757	0.681	0.505
Mediastinal widening	[MED]	0.349	0.092	0.196	0.153
Enlarged hilum	[ENH]	0.387	0.102	0.224	0.19
Tortuous aorta	[TAO]	0.442	0.46	0.39	0.326
Vascular calcification	[VAC]	0.439	0.416	0.362	0.299
Pneumothorax	[PNE]	0.606	0.335	0.46	0.39
Lung lesion	[LLS]	0.410	0.174	0.272	0.242
Mass/nodule	[MAS]	0.349	0.124	0.216	0.187
Hyperaeration	[HYP]	0.512	0.418	0.453	0.435
**Average**	-	**0.454**	**0.285**	**0.345**	**0.285**

2) Multi-Label Pathology Detector: The performance of the pathology detector on standard object detection metrics is presented in Table III. Our model demonstrates strong performance, achieving an overall average precision of 45.4% and a mAP@0.5 of 0.345, effectively identifying all targeted findings. These results underscore the detector's capacity to localize and classify a wide range of radiographic abnormalities with consistent accuracy.

Moreover, the model successfully mitigates the persistent long-tail effect. The most frequent condition, 'Lung opacity', does not exhibit overfitting and achieves both precision and recall above 0.57. Other rare conditions like hyperaeration and pneumothorax also yield competitive results, further validating the detector's generalizability across diverse pathology types.

Despite achieving high precision and mAP for second-level nodes, the recall is generally lower, particularly for prevalent conditions like airspace opacity and consolidation, where recall is below 0.1. This is likely due to their overlap with the highly prevalent 'lung opacity', complicating the differentiation and lowering recall for less distinctive findings. In contrast, conditions unrelated to 'lung opacity', such as hyperaeration (0.59%) and pneumothorax (0.82%), achieve excellent results despite their rarity. Nonetheless, this challenge is unlikely to adversely affect report generation, as the observed detection patterns reflect actual reports, where lung opacity often coexists with these secondary conditions. Therefore, even when secondary nodes are occasionally missed, prompts based on lung opacity and anatomy-level cues remain effective in guiding the decoder.

III. DISCUSSION

A. Limitations and Future Work

While our model demonstrates promising results, several practical considerations remain relevant for future extensions. Currently, the system is designed around image-only inputs and does not explicitly leverage auxiliary multimodal input, such as health records or prior exams and reports. Incorporating such information with careful cross-modal alignment could provide complementary semantic cues and further improve report generation coherence, as suggested by Nicolson et al. [8]. Moreover, the model's performance is influenced by the quality and diversity of the training data, with underrepresented pathologies or rare anatomical anomalies often leading to suboptimal reports. Future work may therefore focus on improving supervision quality and coverage, for example by reducing label noise, enriching region-level annotations, and strengthening cross-institution generalization through more diverse acquisition settings.

Additionally, the language decoder used in this study contains about 2 billion parameters, which may limit its capacity to model complex diagnostic semantics and fully exploit prompt guidance. The recent success of Vision-Language Foundation Models [9]–[11] indicates that using LLMs as report decoders, alongside high-quality and diverse datasets, could yield more nuanced interpretations and clinically detailed reports. However, this would require substantial computational resources and significantly larger datasets to ensure effective fine-tuning.

References

[1] J. Wu, N. Agu, I. Lourentzou, A. Sharma, J. Paguio, J. S. Yao, E. C. Dee, W. Mitchell, S. Kashyap, A. Giovannini *et al.*, "Chest imagenome dataset," *Physio Net*, 2021.

[2] L. Yang, H. Jiang, Q. Song, and J. Guo, "A survey on long-tailed visual recognition," *Int. J. Comput. Vision*, vol. 130, no. 7, pp. 1837–1872, 2022.

[3] L. Yang, S. Xu, A. Sellergren, T. Kohlberger, Y. Zhou, I. Ktena, A. Kiraly, F. Ahmed, F. Hormozdiari, T. Jaroensri *et al.*, "Advancing multimodal medical capabilities of gemini," *arXiv:2405.03162*, 2024.

[4] N. Sharma, "Cxr-agent: Vision-language models for chest x-ray interpretation with uncertainty aware radiology reporting," *arXiv:2407.08811*, 2024.

[5] I. Loshchilov and F. Hutter, "Decoupled weight decay regularization," *arXiv:1711.05101*, 2017.

[6] Ultralytics, "YOLOv5: A state-of-the-art real-time object detection system," 2021. [Online]. Available: https://docs.ultralytics.com

[7] J. Devlin, M.-W. Chang, K. Lee, and K. Toutanova, "BERT: Pre-training of deep bidirectional transformers for language understanding," in *Proc. Conf. North Amer. Chapter Assoc. Comput. Linguistics.*, 2019, pp. 4171–4186.

[8] A. Nicolson, S. Zhuang, J. Dowling, and B. Koopman, "The impact of auxiliary patient data on automated chest X-ray report generation and how to incorporate it," in *Proc. 63rd Annu. Meet. Assoc. Comput. Linguist.*, 2025, pp. 177–203.

[9] Z. Chen, M. Varma, J.-B. Delbrouck, M. Paschali, L. Blankemeier, D. V. Veen, J. M. J. Valanarasu, A. Youssef, J. P. Cohen, E. P. Reis, E. B. Tsai, A. Johnston, C. Olsen, T. M. Abraham, S. Gatidis, A. S. Chaudhari, and C. Langlotz, "Chexagent: Towards a foundation model for chest x-ray interpretation," *arXiv:2401.12208*, 2024.

[10] N. C. F. Codella, Y. Jin, S. Jain, Y. Gu *et al.*, "Medimageinsight: An open-source embedding model for general domain medical imaging," *arXiv:2410.06542*, 2024.

[11] M. Moor, O. Banerjee, Z. S. H. Abad, H. M. Krumholz, J. Leskovec, E. J. Topol, and P. Rajpurkar, "Foundation models for generalist medical artificial intelligence," *Nature*, vol. 616, no. 7956, pp. 259–265, 2023.
